# Metagenome-Assembled Genome Sequence of *Kapabacteriales* Bacterium Strain Clear-D13, Assembled from a Harmful Algal Bloom Enrichment Culture

**DOI:** 10.1128/MRA.01118-20

**Published:** 2020-12-03

**Authors:** Shaikha Al-Saud, Kyra M. Florea, Eric A. Webb, J. Cameron Thrash

**Affiliations:** a Department of Biological Sciences, University of Southern California, Los Angeles, California, USA; Georgia Institute of Technology

## Abstract

Metagenomic sequencing of a *Dolichospermum circinale* enrichment culture resulted in the assembly of several cocultured metagenome-assembled genomes (MAGs). One MAG was affiliated with the class *Kapabacteriales* and included 5,724,991 bp in 127 contigs with a GC content of 48.4%.

## Abstract (precis)

Metagenomic sequencing of a *Dolichospermum circinale* enrichment culture resulted in the assembly of several cocultured metagenome-assembled genomes (MAGs). One MAG was affiliated with the class *Kappabacteriales* and included 5,724,991 bp in 127 contigs, with a GC content of 48.4%.

## ANNOUNCEMENT

Cyanobacterial harmful algal blooms (cyanoHABs) are a significant environmental threat in freshwater environments globally (1), and *Dolichospermum* is a prominent cyanoHAB genus (2), prompting interest in the organisms that constitute these blooms. Metagenomic sequencing and assembly of a *Dolichospermum* enrichment culture yielded a metagenome-assembled genome (MAG) from a cooccurring organism belonging to the class *Kapabacteriales*. We present the details of the sequencing and reconstruction of this MAG to aid in understanding organisms that coexist and potentially interact in cyanoHABs.

We hand-isolated *Dolichospermum* colonies from samples collected via surface tow at Clear Lake, CA (lat 38.973166, long 122.72809), in August 2019. Clear Lake is included on the California 303(d) list of impaired waters because of nutrients and experiences frequent cyanoHABs, including those formed by the cyanobacterial genus *Dolichospermum* (3). We identified *Dolichospermum* colonies morphologically (4) using an Axiostar epifluorescence microscope (Zeiss, Oberkochen, Germany). Multiple colonies were cultured in 50% BG-11_0_ medium at 25°C and 100 μmol Q/m^2^/s on a 12:12-h light/dark cycle for roughly 7 months. We maintained growth by adding medium every 2 weeks. BG-11_0_ medium lacks a nitrogen source in order to selectively grow diazotrophs. To isolate DNA, we filtered 50 ml of a single enrichment culture onto 25-mm-diameter 8-μm polycarbonate filters. The collected solids were then rinsed into 2-ml bead-beating tubes using the lysing solution from the DNeasy PowerBiofilm kit (Qiagen, Hilden, Germany). We subjected the tubes to five freeze-thaw cycles in liquid nitrogen, followed by an overnight incubation in a proteinase K solution (25 μl of a 25-mg/ml stock solution) at 55°C to induce cell lysing. Genomic DNA was then extracted via the Qiagen PowerBiofilm kit following the manufacturer’s instructions. DNA was quantified with NanoDrop UV-visible (UV-Vis) spectroscopy and Qubit spectrofluorometry (Thermo Fisher Scientific, Waltham, MA). Illumina 150-bp paired-end (PE) sequencing (1 Gbp) with 300-bp inserts was performed by Novogene (Nanjing, China) after library preparation with a NEBNext DNA library preparation kit according to the manufacturer's recommendations. This resulted in 19,844,532 reads. We performed quality control with FastQC v0.11.5 (5) and Trimmomatic v0.36 (6), assembly with metaSPAdes v3.13.0 (7), and binning with MaxBin v2.2.4 (8) on KBase (9). The genome was annotated via PGAP (10). We completed taxonomic identification with GTDB-tk v1.1.1, run with “classify_wf” using the release 95 database. Functional annotation was estimated using FuncSanity as part of the MetaSanity v1.2 wrapper (11). Default settings were used for all software unless otherwise noted.

Clear-D13 had 5,724,991 bp in 127 contigs, an *N*_50_ value of 90,289 bp, and a GC content of 48.4%. CheckM v1.0.18 (12) estimated the MAG as 96.2% complete with 2% contamination. The genome is predicted to contain 4,434 genes. GTDB-tk identified Clear-D13 as belonging to the class *Kapabacteriales* (formerly *Ignavibacteria*), in the provisional genus PH2015. Genome analysis revealed that this strain contains a cluster for sulfur assimilation and metabolism, including sulfite dehydrogenase and sulfide oxidation, which indicates putative chemolithotrophic capabilities. Since no carbon fixation pathways were observed, this organism is likely heterotrophic. Transporters for cobalt (*corA*), copper (*copA*), ferrous iron (*feoB*), Fe-Mn (*mnth*), phosphate (*pst*), phosphonate (*phn*), and ammonia (*amt*) were also identified. The presence of the glyoxylate shunt pathway indicates that this MAG yields the potential to use small carbon sources like acetate.

### Data availability.

This whole-genome shotgun project has been deposited at DDBJ/ENA/GenBank under the accession number JACVZY000000000. The version described in this paper is version JACVZY010000000. The BioProject number is PRJNA657201, and the reads are available in the SRA under the accession number SRX8961729.
